# Optimization of Culture Conditions for High Cell Productivity and Astaxanthin Accumulation in Vietnam’s Green Microalgae *Haematococcus pluvialis* HB and a Neuroprotective Activity of Its Astaxanthin

**DOI:** 10.3390/bioengineering11121176

**Published:** 2024-11-21

**Authors:** Nguyen Cam Ha, Luu Thi Tam, Hoang Thi Minh Hien, Ngo Thi Hoai Thu, Dang Diem Hong, Le Thi Thom

**Affiliations:** 1 Institute of Biotechnology, Vietnam Academy of Science and Technology (VAST), Hanoi 10000, Vietnam; hanguyen.hou@gmail.com (N.C.H.); tamluu5458kl@gmail.com (L.T.T.); hmhien@ibt.ac.vn (H.T.M.H.); nhthu@ibt.ac.vn (N.T.H.T.); ddhong60vn@yahoo.com (D.D.H.); 2 Faculty of Biotechnology, Graduate University of Science and Technology, Vietnam Academy of Science and Technology (VAST), Hanoi 10000, Vietnam

**Keywords:** astaxanthin, bicarbonate, *Haematococcus pluvialis*, perfusion culture, neuroprotective potential

## Abstract

Background: *Haematococcus pluvialis*, a green microalga, is a rich source of natural astaxanthin and a potent antioxidant with high commercial value. This study investigates the biological characteristics and potential of *H. pluvialis* HB isolated from Hoa Binh, Vietnam, for growth and astaxanthin accumulation using a two-phase culture method. Methods: *H. pluvialis* HB was cultured in a C/RM medium at 25 °C, and morphological characteristics were examined. NMR spectroscopy was used to determine the structure of the astaxanthin, which was extracted using the Soxhlet method. Results: After 22 days, the highest cell density (4.96 × 10^6^ cells mL^−1^) was achieved under optimized light and ultraviolet conditions. Nutrient deprivation followed by bicarbonate supplementation resulted in a maximal astaxanthin accumulation of 48.8 mg g^−1^ dry cell weight within two days. The extracted astaxanthin demonstrated potent antioxidant activity (IC_50_: 3.74 mg mL^−1^) compared to ascorbic acid (IC_50_: 18.53 µg mL^−1^) and exhibited strong acetylcholinesterase inhibition (IC_50_: 297.99 µg mL^−1^). It also showed neuroprotective effects against H_2_O_2_ and amyloid beta-induced neurotoxicity in C6 cells. Conclusions: This study highlights *H. pluvialis* HB as a promising source for large-scale astaxanthin production with potential applications in neuroprotective health products.

## 1. Introduction

The unicellular freshwater microalga *Haematococcus pluvialis* (Chlorophyceae) is widely recognized as the primary natural source of astaxanthin, a carotenoid with a variety of applications. *H. pluvialis* can accumulate astaxanthin up to 5% of its dry cell weight (DCW), the highest concentration reported among naturally occurring astaxanthin-producing organisms [1]. In the two-stage production process, enhancing cell density during the growth phase and optimizing critical factors for astaxanthin accumulation in the induction phase are the keys to improving production efficiency in *H. pluvialis*. Studies have shown that optimizing nutrients in the culture medium, such as nitrate (KNO_3_, NaNO_3_) and vitamins, can significantly boost the productivity of vegetative *Haematococcus* cultures [2].

Currently, different astaxanthin extraction methods, including using various organic solvents, enzyme lysis, acid/base treatments, and supercritical CO_2_, have been investigated. However, disadvantages such as toxicity, high pressure, and the need for pre-treatment drying limit the use of such methods. Kang and Sim (2008) and Yazgin et al. (2020) showed extraction with vegetable oils that are eco-friendly, simple, economical, and do not require high amounts of energy [3,4].

A simple and cost effective method for cultivating *H. pluvialis* (CCAP 34/1D) with a cell density of 4.98 × 10^5^ cells mL^−1^, using urea as a nitrogen source in the initial stage, was reported by Rizzo et al. [5]. The highest cell density achieved, 1 × 10^6^ cells mL^−1^, was observed in a 500 mL flask [6]. *H. pluvialis* has also been cultivated in various bioreactors, including bubble columns and airlift reactors. For instance, Wan et al. [7] demonstrated successful heterotrophic cultivation of *H. pluvialis* ZY-18 in fed-batch mode using a 50 L fermenter, achieving a high cell density of 7 × 10^6^ cells mL^−1^ after 405 h. In contrast, cell density was lower in stirred tank reactors and open ponds, reaching only 29 × 10^4^ cells mL^−1^ [8].

Astaxanthin is a carotenoid with a market value exceeding USD 200 million per year [9]. Natural astaxanthin has demonstrated advantages over its synthetic counterpart in both structure and function [10]. Firstly, synthetic astaxanthin is cheaper than natural microalgal astaxanthin since microalgal cultivation and harvesting are cost consuming. Secondly, synthesis astaxanthin is completely different from natural astaxanthin that comprises 95% esterified molecules. Thirdly, the forms of astaxanthin contain different geometrical and optical isomers. According to Capelli et al. [10], natural astaxanthin is over 50 times stronger than synthetic astaxanthin in singlet oxygen quenching and approximately 20 times stronger in free radical elimination. Furthermore, synthetic astaxanthin is markedly inferior to algal natural astaxanthin as an antioxidant, which makes it possible to have a huge health benefit and help prevent various diseases [10,11]. It has been shown to have neuroprotective, cardiovascular, and skin-protective properties, as well as various pharmacological effects, including anti-cancer, anti-diabetic, anti-inflammatory, and antioxidant activities [12]. Carotenoids, such as astaxanthin, inhibit H_2_O_2_-induced oxidative stress and offer protective effects through anti-inflammatory and anti-apoptotic mechanisms [12,13].

Astaxanthin synthesis in *H. pluvialis* is triggered by unfavorable environmental conditions such as high light intensity, elevated temperature, increased salinity, nutrient depletion, and the presence of plant hormones, which inhibit cell division and growth. In one study, astaxanthin levels increased from 0.313 ± 0.008 to 0.711 ± 0.143 mg g^−1^ when 0.25% (*w*/*v*) sodium acetate was added and irradiance was increased [5]. A study in Vietnam demonstrated that after 10 days of cultivation in pilot-scale, angled twin-layer porous substrate photobioreactors (PBRs) with red/blue LED light, *H. pluvialis* reached a maximum DCW of 40.74 g m^−2^, with an astaxanthin content of 1.3% (*w*/*w*) [14]. A previous study reported the successful design and operation of biofilm-based PBR systems for *H. pluvialis* cultivation at both small (0.05 m^2^) and large (2 m^2^) scales, achieving DCW productivity of 12 g m^−2^ d^−1^ (3% astaxanthin) and 11.25 g m^−2^ d^−1^ (2.8% astaxanthin), respectively, after 10 days [15]. Numerous investigations have been carried out to determine the optimal technique for growing *H. pluvialis* microalgae with a not very high astaxanthin content, including photoautotrophic and heterotrophic approaches. The results showed that under cultivating heterotrophically (using acetate as a C source), the astaxanthin content was 3–4 times lower than photoautotrophically in a nitrogen-deficient medium supplemented with bicarbonate (HCO_3_^−^) or CO_2_ continuously and irradiated with high-intensity light [16]. Currently, cultivating *H. pluvialis* using a two-phase cultivation process has achieved many outstanding results. In Vietnam, in a recent study by Vinh (2023) [17], two-phase cultivation of *H. pluvialis* in an RM medium with stress conditions causing astaxanthin accumulation for four consecutive days was carried out. On a 20 L scale, phase I used an RM liquid medium, white LED light (light intensity of 90 μmol photons m^−2^ s^−1^), a lighting time of 12 h, continuous aeration, and 118 ppm of CO_2_. In phase II, light intensity was increased to 120 μmol m^−2^ s^−1^, leading to an astaxanthin content increase from 5144 to 7535.8 μg L^−1^, accounting for 2.34–2.61% DCW. In the cultivation of *H. pluvialis*, high light or nutrient depletion both contribute to the growth and accumulation of astaxanthin [17]. Huy et al. (2022) reported that the carotenoid content of *H. pluvialis* cultured in a BG-11 medium under nitrate reduction inhibition and NPK supplementation reached its highest value of 54.709 mg g^−1^ [18]. A publication by Trung et al. (2021) also showed that the low light intensity of 20 to 50 μmol photons m^−2^ s^−1^ is suitable for the vegetative culture stage of *H. pluvialis* cells; meanwhile, a high light intensity of 70 to 100 μmol photons m^−2^ s^−1^ stimulates cyst formation and increases the synthesis of pigments and antioxidant capacity of *H. pluvialis* cells [19]. The experimental results by Ngoc et al. (2022) showed that the highest cell density was obtained at 19.3 × 10^4^ cell mL^−1^ with a specific growth rate of 0.05/day in the CMS medium 0.2%. At a low concentration of 0.01% CMS, the highest accumulative astaxanthin concentration was found up to 3.1% DCW. It indicated that the faster the astaxanthin accumulation time, the lower the CMS concentration [20]. But, all these above-mentioned reports indicated that this approach was not feasible and effective for large-scale astaxanthin synthesis.

Critical reviews of the literature identified a significant challenge in astaxanthin production. The 10-day red cell turnover time is lengthy, leading to a loss of microalgae and hindering practical large-scale application [21,22]. Optimizing culture conditions to enhance cell productivity is essential to overcoming this limitation. Furthermore, astaxanthin was extracted from *H. pluvialis* HB biomass and evaluated for its bioactivity, specifically its neuroprotective potential [21,22]. This included testing its antioxidant properties, acetylcholinesterase (AChE) inhibitory activity, and its ability to protect against cytotoxicity in an in vitro C6 cell model of Alzheimer’s disease (AD) induced by H_2_O_2_ or Aβ_25–35_. To date, such investigations on the neuroprotective properties of astaxanthin from *H. pluvialis* HB have not been published. Therefore, we conducted this study to investigate the biological characteristics and assess the growth and astaxanthin accumulation potential of *H. pluvialis* HB, a strain isolated from Hoa Binh province, Vietnam, using a two-phase culture technique. The aim of this study is to obtain conditions for cultivating *Haematococcus pluvialis* HB rich in astaxanthin, which has antioxidant and neuroprotective effects, as a food to protect human health.

## 2. Materials and Methods

### 2.1. Microalgae Cultivation

The microalgae *Haematococcus pluvialis* HB, isolated from freshwater lakes in Hoa Binh province, Vietnam, and deposited at the Algal Biotechnology Department, Institute of Biotechnology, Vietnam Academy of Science and Technology (VAST), with accession number HPHB01, was used in this study. The strain was cultured in 250 mL Erlenmeyer flasks containing a C medium under white light, a visible light wavelength in the range of 380–760 nm, and continuous illumination of 40 µmol photons m^−2^ s^−1^ with a 12:12 h light/dark photoperiod at 25 ± 2 °C.

### 2.2. Cell Culture and Treatment

C6 rat glial cells (ATCC, CCL-107™) were cultured in Dulbecco’s Modified Eagle Medium (DMEM) (Merck, Darmstadt, Germany)/high glucose, supplemented with 10% fetal bovine serum (FBS) and 1% penicillin/streptomycin, at 37 °C with 5% CO_2_. LPS and Aβ were used to differentiate the cells into disease-specific phenotypes, such as those mimicking Alzheimer’s disease [23].

*H. pluvialis* HB was grown on agar or C/RM medium at 25 °C. Morphological characteristics and life cycle stages were observed using a light microscope (Olympus CX21, Osaka, Japan) and a scanning electron microscope (SEM, model JSM-5410L; Jeol Company, Tokyo, Japan). Cell size was measured using Mapinfo 7.5 software [24].

Dry cell weight (DCW) and cell density were used to measure growth. Samples were harvested from a 15 mL culture broth, centrifuged at 5000× *g* for 5 min, dried at 105 °C, and weighed. Cell density was determined using a Burker–Turk counting chamber (Hirschmann, Laborgeräte Hilgenberg, Germany) [16].

Pigments were analyzed spectrophotometrically using the method described by Strickland and Parson (1960). Total carotenoids were extracted with 90% acetone and expressed as astaxanthin based on thin-layer chromatography results [25].

The specific growth rate (µ) was calculated according to the method described by Imamoglu et al. [26].

Protein content was measured using the Lowry method, with bovine serum albumin as a standard [27].

### 2.3. Effect of Different Culture Media on Cell Growth

The microalgae were cultivated in four different media: C, modified BG-11, OHM, and RM. Cultures were maintained in 250 mL Erlenmeyer flasks with a 150 mL working volume, illuminated at 40 µmol photons m^−2^ s^−1^ with a 12:12 h light cycle at 25 ± 2 °C. Initial cell density was set at 6 × 10^4^ cells mL^−1^, with samples collected every two days to measure cell density, growth rate, and pigment content. Each experimental condition was performed in triplicate.

### 2.4. Combined Effects of Lighting Regime, Nitrate Concentration, and Cultivation Model on Cell Growth

Microalgae were cultured in 10 L plastic bottles containing 4 L RM medium with an initial cell density of 0.5 × 10^6^ cells mL^−1^, continuous aeration, and a temperature of 25 ± 0.5 °C. Three experimental conditions were evaluated.

Control. The RM-4X medium with nitrate concentration was four times that of the RM medium, illuminated by a fluorescent lamp at 50 µmol photons m^−2^ s^−1^ with a 12:12 h light photoperiod.

Experiment 1. The RM-4X medium illuminated at 85 µmol photons m^−2^ s^−1^ with a 16:8 h light photoperiod.

Experiment 2. High light (85 µmol photons m^−2^ s^−1^) was combined with UV light (30 µmol photons m^−2^ s^−1^) and a 16:8 h light photoperiod.

The light regime was implemented in the following order: 5 h of white high light, 6 h of white high light mixed with UV light, and finally, 5 h of white high light. When the culture volume has reached the maximum (5 L) compared to the culture bottle, the perfusion culture process is carried out under continuous aeration conditions (aeration rate is 0.5 L min^−1^).

### 2.5. Extraction of Astaxanthin from H. pluvialis HB by the Soxhlet Method

The Soxhlet method as published by Hien et al. [28] was used to extract astaxanthin (HA1 compound) directly from the dry biomass for 3 h with an extraction solvent of dichloromethane. The extraction solution was concentrated by a vacuum distillation rotary evaporator. The astaxanthin was then gradually precipitated by the addition of ethanol, after which the residue was further dissolved in dichloromethane and crystallized in ethanol. The extracted astaxanthin (compound HA1 was obtained as a red amorphous powder) had 95% purity, as confirmed by HPLC, with a 4.5% DCW content. NMR spectroscopy was used to determine the structure of the astaxanthin. On the 1H-NMR spectrum in the strong field region, four characteristic signals of methyl groups appear at δ_H_ 1.32 (3H, s, H-16), 1.21 (3H, s, H-17), 1.94 (3H, s, H-18), 2.00 (3H, s, H-19), and 1.99 (3H, s, H-20). Additionally, there are two signals of the methylene group [δ_H_ 2.15 (1H, dd, 12.6, 6.0 Hz, H-2, and 1.81 (1H, dd, 13.8, 12.6 Hz, H-2)] and a signal of the methine oxygen group at δ_H_ 4.32 (1H, dd, 13.8, 6.0 Hz, H-3). Seven olefin proton signals appear in the range δH 6.20–6.67 towards the low field region. The NMR spectra were in agreement with the published research [29].

### 2.6. Influence of Nutrient Conditions on Astaxanthin Production and Its Bioactivity in Microalgae

During the exponential growth phase, microalgae cells were subjected to nutrient deprivation and various bicarbonate concentrations (60, 80, 100, 120, and 160 mM) to induce astaxanthin accumulation under constant aeration (0.5 L min^−1^) and high light conditions (85 µmol photons m^−2^ s^−1^) with a 16:8 h light/dark photoperiod. Each experimental condition was performed in triplicate.

Astaxanthin was extracted from the dry biomass using the Soxhlet method with dichloromethane solvent [28]. The extracted astaxanthin was confirmed to have 95% purity by HPLC.

The antioxidant activity of astaxanthin was assessed using the DPPH assay, as described by Hien et al. (2023) [28]. A total of 100 mL of a 0.2 mM DPPH solution in methanol was mixed with 100 mL of astaxanthin at 10–500 mg mL^−1^, followed by a 30 min dark incubation period at room temperature. Absorbance was measured at 517 nm, and ascorbic acid was used as a positive control. The determination of antioxidant activity is expressed in IC_50_ (μg mL^−1^) as antioxidant capacity. The IC_50_ value is defined as the concentration of test compounds that can inhibit free radicals by as much as 50%. The smaller the IC_50_ value, the higher the free radical reduction activity [28].

The acetylcholinesterase (AChE) inhibitory activity of astaxanthin was measured using the Acetylcholinesterase Inhibitor Screening Kit (MAK324, Sigma, St. Louis, MO, USA) with galantamine with 4, 20, 100, and 500 µg mL^−1^ concentrations as a positive control.

C6 cells were treated with H_2_O_2_-induced oxidative stress or Aβ_25–35_-induced cytotoxicity in the presence of astaxanthin (50, 100, and 200 µg mL^−1^) or positive controls, such as ascorbic acid and galantamine. Cell viability was assessed using the MTT assay.

Cell viability was evaluated using the MTT assay, as described by Hien et al. [28].

### 2.7. Statistical Analysis

All experiments were performed in triplicate, and data were presented as the mean ± standard error of the mean (SEM). Statistical significance was determined using Student’s *t*-test, with *p* < 0.05 considered statistically significant.

## 3. Results

### 3.1. Morphological Changes in the Life Cycle of H. pluvialis Strain HB

**Figure 1:**
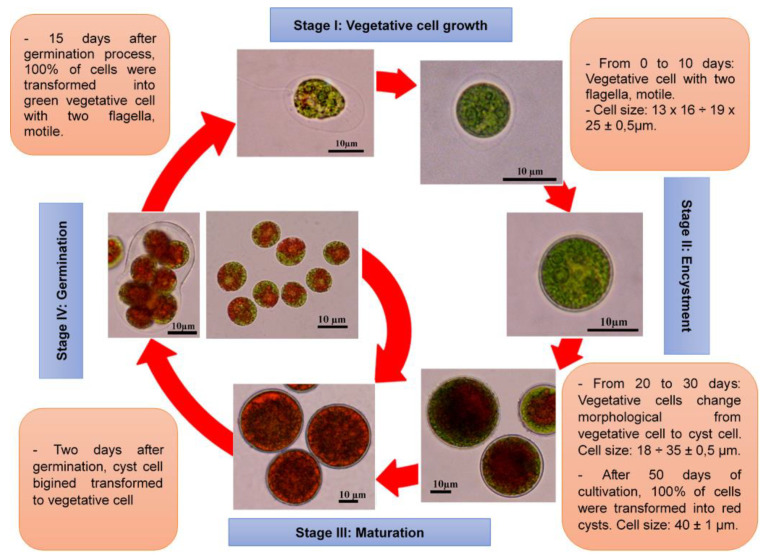
Schematic diagram of the life cycle model of *H. pluvialis* HB. (1) Stage I: vegetative cell growth (lasting from day 0 to day 10); (2) stage II: encystment (from day 10 to day 40); (3) stage III: maturation (from day 40 to day 50); and (4) stage IV: germination (occurring within 2 days)).

**Figure 2:**
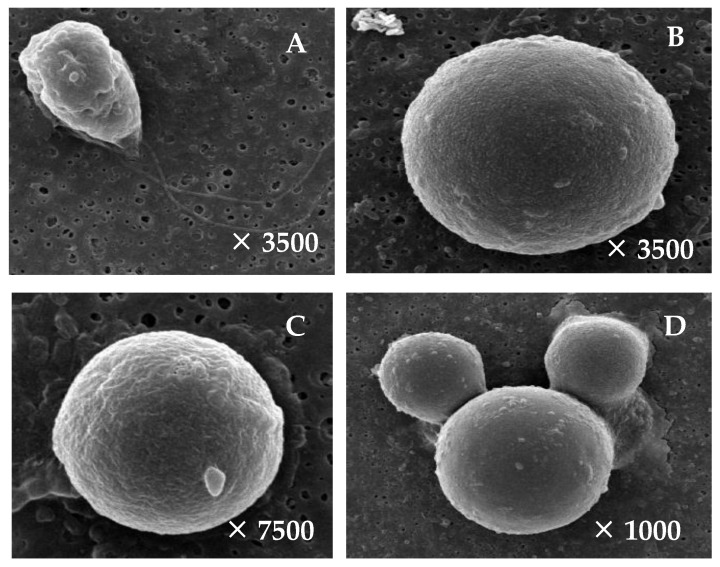
Images of the *H. pluvialis* HB cell at different stages under a scanning electron microscope (SEM, magnification shown in each picture). (**A**) Cells in vegetative growth in stage I. (**B**) Cells in encystment in stage II. (**C**) Cells in maturation in stage III. (**D**) Cells in germination in stage IV.

Figure 1 and Figure 2 depict the schematic representation of the life cycle model and the scanning electron microscope (SEM) images of *H. pluvialis* strain HB cells at different stages of development. The 50-day life cycle of strain HB in the RM medium is divided into four distinct stages.

There are four morphological forms of *H. pluvialis* microalgae cells observed during the life cycle. The vegetative cells have green ellipticals and two flagella and are capable of movement. This cell accounted for 90% of the total number of cells in the first 10 days of cultivation, with a size ranging from 13 × 16 ÷ 19 × 25 μm. From 10 to 20 days of cultivation, there is a gradual decrease in vegetative cells and an increases in encyst cells that are spherical, green in color, lost flagella, and are not able to be motile. After 20 days of cultivation, the cells completely changed to encyst form, increased their size significantly (reaching about 40 μm), and began to accumulate astaxanthin. After 50 days of culture, 100% of the cells were transformed into red cysts with a dark red color, a thick cell wall, and astaxanthin as the main pigment. The germination stage is calculated from the time of transferring cyst cells into the new medium by centrifuging to collect cell residue. This stage lasted for 2 days. In the first 24 h, there is a change in the color of the internal substance inside the cell from dark red to reddish brown. In the next 24 h, there is the actual germination of the cells.

**Figure 3:**
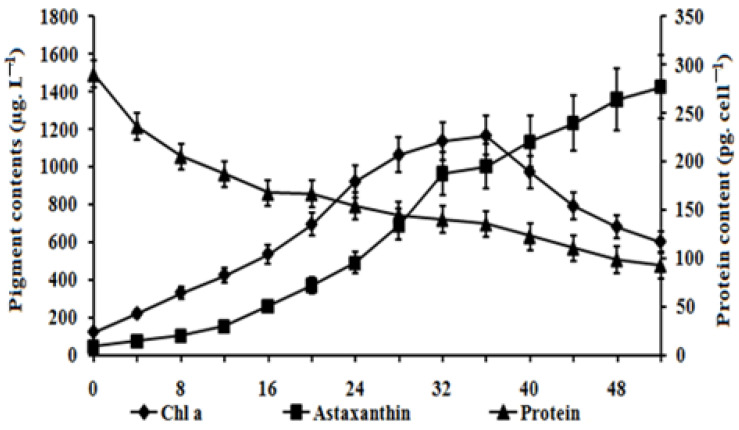
The pigment contents (chlorophyll a, astaxanthin) and protein content of *H. pluvialis* HB in the RM medium.

The cell density of strain HB reached 60 × 10^4^ cells mL^−1^ after 40 days of cultivation (Supplementary Figure S1). Chlorophyll content increased during the first 36 days of cultivation but declined in the subsequent days. In contrast, astaxanthin content steadily increased, peaking at approximately 1.424 µg mL^−1^ by day 50. As cells transitioned from the vegetative to the cyst stage, with an accumulation of astaxanthin, intracellular protein levels exhibited a downward trend (Figure 3). Protein content decreased approximately 20-fold by day 50, falling below 100 pg cell^−1^ compared to the vegetative stage. During stages II and III, *H. pluvialis* cells lost their flagella and motility, and the cell diameter increased dramatically from 10–20 µm to 40–50 µm (Figure 1 and Figure 2).

Our findings also indicated that the astaxanthin/chlorophyll ratio varied across the stages: 0.30 ± 0.05 in the vegetative stage, 0.90 ± 0.20 during encystment, 2.40 ± 0.10 in the cyst stage, and 0.60 ± 0.20 during germination.

### 3.2. Determination of Optimal Culture Conditions for the Growth of H. pluvialis Strain HB

#### 3.2.1. Selection of the Optimal Culture Medium for Strain HB

**Figure 4:**
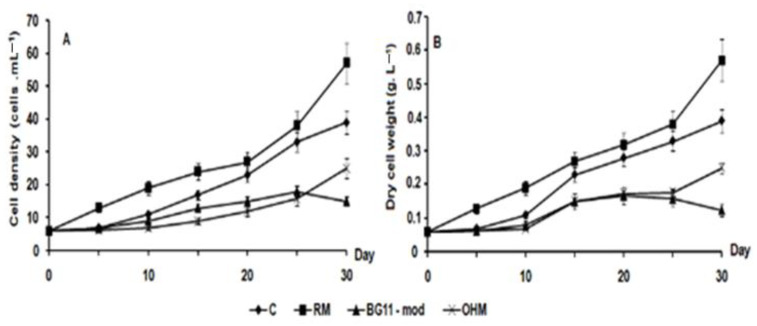
Cell density (**A**) and dry cell weight (**B**) of *H. pluvialis* HB cultured in the different media C (⧫), modified BG-11 (▪), OHM (▲), and RM (×) in 250 mL Erlenmeyer flasks.

The RM medium proved to be the most effective for algal cultivation, achieving a maximum cell density of 5.74 × 10^5^ cells mL^−1^, dry cell weight (DCW) of 0.511 g L^−1^, and a specific growth rate (μ) of 0.062/day after 30 days (Figure 4). To extend the vegetative growth phase (stage I), it is essential to transition the culture from test tubes to 250 mL Erlenmeyer flasks, adding fresh medium to 50% of the culture volume at intervals of 2 to 4 days (Figure 1).

#### 3.2.2. Combined Effects of Illumination, Nitrate Concentration, and Cultivation Model on Cell Growth

**Figure 5:**
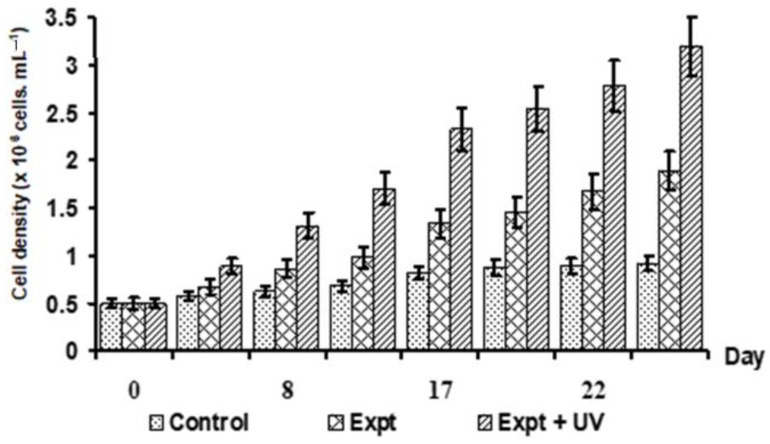
Cell density of *H. pluvialis* HB at different illumination conditions. Control: the microalga was cultured in the RM—4X medium (contained nitrate concentration four times higher than the basal medium of RM) under an illumination of 50 µmol photons. m^−2^ s^−1^ provided by white fluorescent light with a light/dark 12:12 h photoperiod; experiment (Expt.): cultured the microalga in the RM—4X medium under an illumination of 85 µmol photons. m^−2^ s^−1^ with a light/dark 16:8 h photoperiod; experiment (Expt. + UV): the illumination condition combined ultraviolet (UV). The microalga was cultured in the RM—4X medium combined with a white high light intensity of 85 µmol photons. m^−2^ s^−1^ provided by fluorescence lamps and UV illumination (at 30 µmol photons. m^−2^ s^−1^ provided by UV lamps) and a light/dark 16:8 h photoperiod (including light regime order as 5 h of white high light intensity, 6 h of white high light intensity combined UV light, and finally, 5 h of white high light intensity).

**Figure 6:**
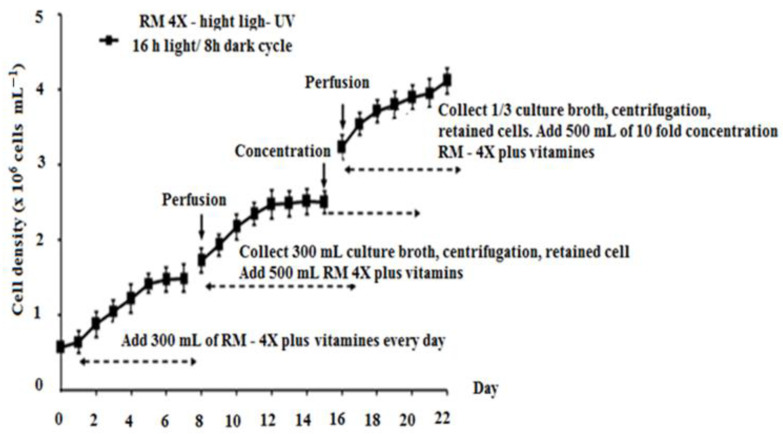
Cell density of *H. pluvialis* HB cultured on the RM—4X medium under lighting conditions with a light/dark cycle of 16:8 h, of which 10 h are illuminated by fluorescent lamps (with an intensity of 85 µmol photons. m^−2^ s^−1^), 6 h of fluorescent illumination combined with UV light (with an intensity of 30 µmol photons. m^−2^ s^−1^), and a perfusion culture procedure.

The combined influence of nitrate concentration and light regime on maximizing vegetative cell density was also investigated. In Figure 5, the highest vegetative cell density (3.2 × 10^6^ cells mL^−1^) was achieved under a 16:8 h light/dark cycle after 22 days of cultivation. The illumination conditions consisted of 10 h of high-intensity white light (85 µmol photons m^−2^ s^−1^) and 6 h of high-intensity white light combined with UV irradiation (30 µmol photons m^−2^ s^−1^). Maximum cell densities of 0.9 × 10^6^ cells mL^−1^ and 1.8 × 10^6^ cells mL^−1^ were observed after 19 days under continuous white light at intensities of 50 µmol photons m^−2^ s^−1^ and 85 µmol photons m^−2^ s^−1^ for 12 and 16 h per day, respectively. Astaxanthin content peaked at 3.200 µg L^−1^ after 13 days of culture (Supplementary Figure S2). To enhance cell density in the vegetative stage, an RM—4X medium (with a tenfold higher concentration) was added using a fed-batch approach after 16 days of cultivation. In Figure 6, the density of vegetative cells significantly increased using the experimental formula (Expt. + UV) in combination with the control (RM—4X medium). A maximum cell density of 4.96 × 10^6^ cells mL^−1^ was achieved after 22 days.

#### 3.2.3. Effects of Bicarbonate (HCO_3_^−^) Concentration on Astaxanthin Accumulation

**Figure 7:**
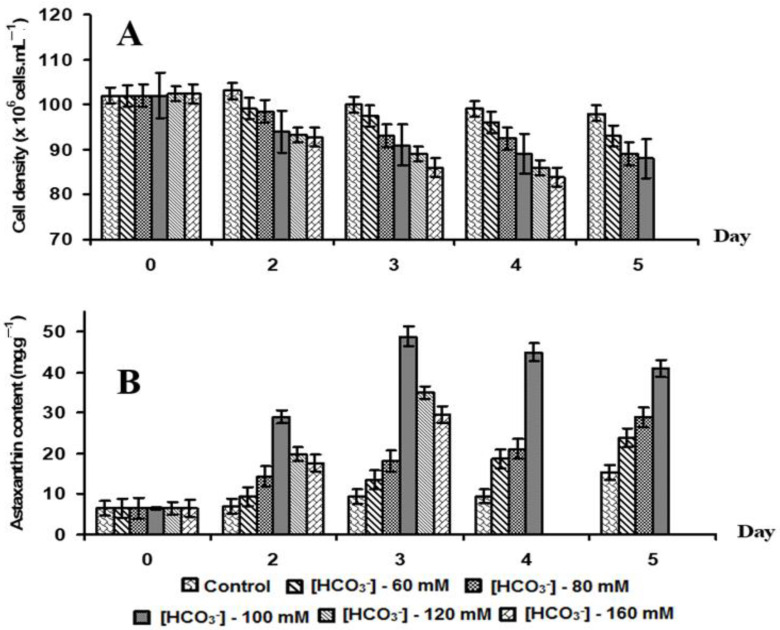
Effect of difference bicarbonate concentration on the growth (**A**) and astaxanthin content (**B**) of microalga *H. pluvialis* HB.

When induced with varying bicarbonate concentrations, algal cell density tended to decrease (Figure 7A). Successful trials conducted at various scales (20, 50, and 100 L), including closed tubular PBRs, demonstrated that bicarbonate was an essential factor for converting green vegetative cells of *H. pluvialis* HB into red cyst cells (Supplementary Figures S3–S5). In Figure 7B, higher bicarbonate concentrations resulted in increased astaxanthin accumulation in red cyst cells. Astaxanthin content rose from 6 to 29 mg g^−1^ DCW when bicarbonate concentration increased from 0 to 80 mM, although the induction period was extended to 5 days.

Raising bicarbonate concentration from 100 to 160 mM over two days of induction significantly increased astaxanthin content, with all green vegetative cells turning into red cysts. The maximum astaxanthin content (48.8 mg g^−1^ DCW) was achieved at 100 mM bicarbonate, an eightfold increase compared to cells without bicarbonate. However, when bicarbonate exceeded 160 mM, the high alkalinity inhibited algal growth and astaxanthin accumulation. The cultivation of *H. pluvialis* HB was carried out using a two-phase culture process. In the first phase, the HB strain was grown under optimal conditions for growth; pH reached 7.0 using the RM-4X medium. In the second phase, the HB strain was subjected to stress conditions to accumulate astaxanthin by adding bicarbonate at a concentration of 100 mM to the culture medium, leading to a pH increase to 9.0. Our efforts resulted in preventing cell replication and increasing astaxanthin production in *H. Pluvialis*, adding a concentration of NaHCO_3_ combined with stress conditions, such as high pH and nitrogen depletion.

### 3.3. Astaxanthin Extraction from H. pluvialis Biomass

**Figure 8:**
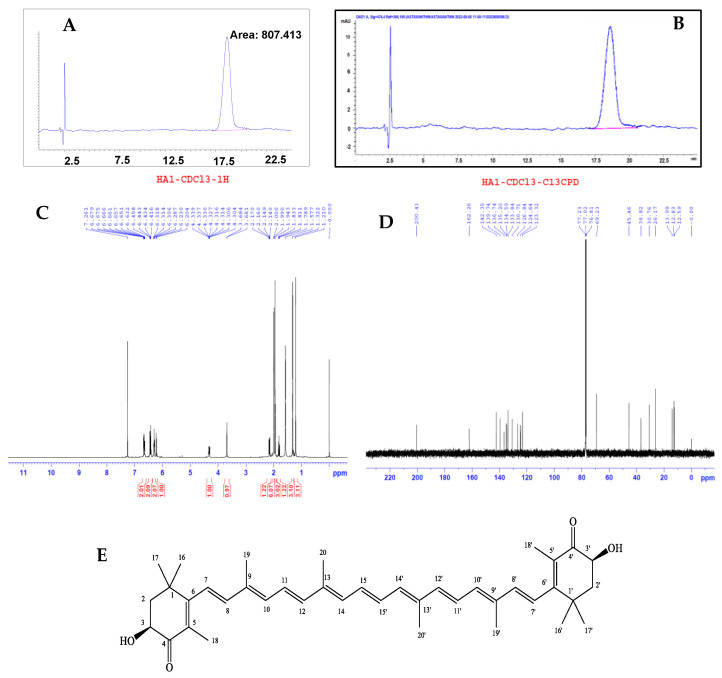
Astaxanthin is extracted from the *H. pluvialis* HB biomass. HPLC chromatograms of standard astaxanthin (**A**) and astaxanthin isolated from *H. pluvialis* HB (**B**). The 1H NMR spectrum of extracted astaxanthin (**C**). The 13C-NMR spectrum of extracted astaxanthin (**D**). The chemical structure of extracted astaxanthin (**E**).

From 100 g of dry *H. pluvialis* HB biomass, 4.5 g of astaxanthin with 95% purity were isolated. Structural confirmation, as shown in Figure 8, demonstrated the high purity of the extracted astaxanthin. The analysis results were measured at the Institute of Chemistry, Vietnam Academy of Science and Technology, Hanoi, Vietnam. Antioxidant activity, acetylcholinesterase (AChE) inhibitory activity, and the protective effects on C6 nerve cells were also evaluated when cells were induced by H_2_O_2_ and protein Aβ_25–35._

### 3.4. Antioxidant Properties of Astaxanthin

Astaxanthin demonstrated significant antioxidant activity. The DPPH assay revealed a 29.87 ± 0.54% radical scavenging capability at a concentration of 2 mg mL^−1^ (Table 1).

#### Acetylcholinesterase (AChE) Inhibitory Activity of Astaxanthin

The AChE inhibitory activity was high (IC_50_ value of 297.99 ± 5.23 µg mL^−1^) compared with positive control galantamine (IC_50_ value of 4.11 ± 0.25 µg mL^−1^) (Table 2). The astaxanthin was a moderate AChE inhibitor.

### 3.5. Cytotoxic Effect of Astaxanthin on C6 Cells

**Figure 9:**
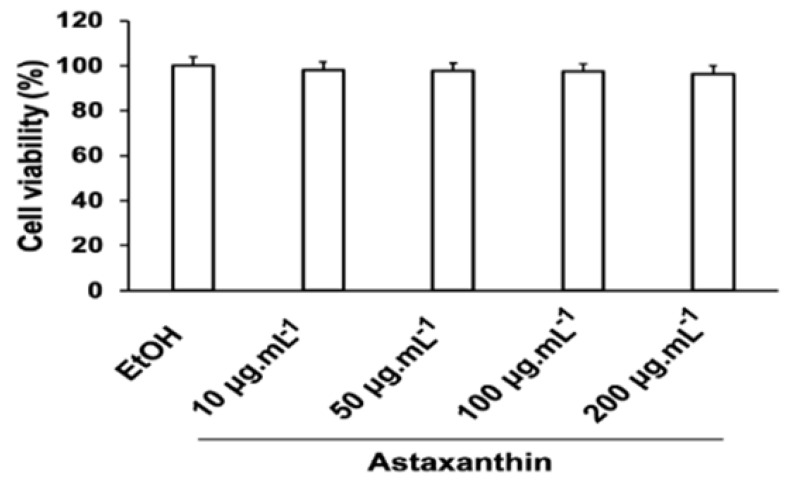
Effect of astaxanthin on the survival of C6 cell lines. Ethanol (EtOH) was used as the control group. Cell viability was assessed by an MTT assay. The data are expressed as the mean ± SEM (n = 3).

The extracted astaxanthin did not exhibit cytotoxicity at the tested concentrations (Figure 9). C6 cell viability remained above 96% after 24 h of incubation with astaxanthin at concentrations of 10, 50, 100, and 200 µg mL^−1^, demonstrating that astaxanthin is non-toxic to C6 cells within this range.

### 3.6. Neuroprotective Effects of Astaxanthin Against Oxidative Stress-Induced Damage in C6 Cells

**Figure 10:**
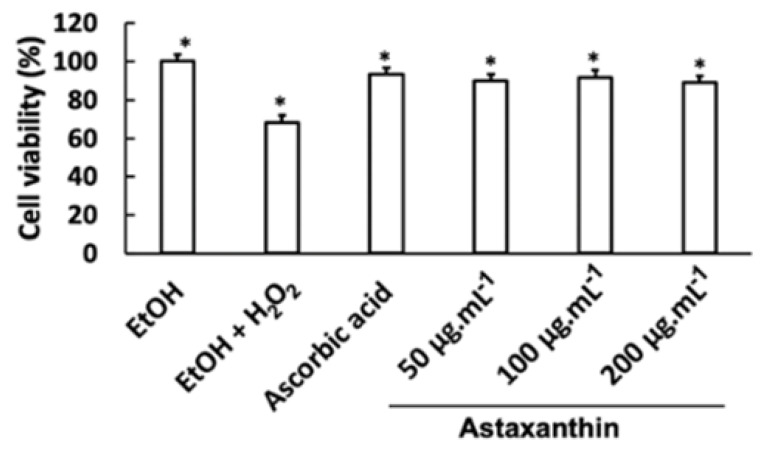
The astaxanthin protected C6 cells against damage by oxidative stress induced by H_2_O_2_ (10 mM). Cells were pre-incubated with astaxanthin (at 50, 100, and 200 µg mL^−1^) or ascorbic acid (20 µg mL^−1^) at the indicated concentration for 24 h prior to 10 mM H_2_O_2_ exposure for 1 h. Cell viability was assessed by an MTT assay. The data are expressed as the mean ± SEM (n = 3). Significant differences in the cell damage induced by H_2_O_2_ are denoted by * *p* < 0.05. EtOH: ethanol; H_2_O_2_: hydrogen peroxide.

Exposure of C6 cells to 10 mM H_2_O_2_ resulted in a 68.12% reduction in cell viability. However, pre-treatment with ascorbic acid or astaxanthin at concentrations of 50, 100, and 200 µg mL^−1^ significantly increased cell viability to 93.37%, 90.12%, 91.72%, and 89.17%, respectively (Figure 10). These findings suggest that astaxanthin effectively protects C6 cells from oxidative stress-induced cell death caused by H_2_O_2_.

### 3.7. Protective Effects of Astaxanthin Against Aβ_25–35_-Induced Cytotoxicity in C6 Cells

**Figure 11:**
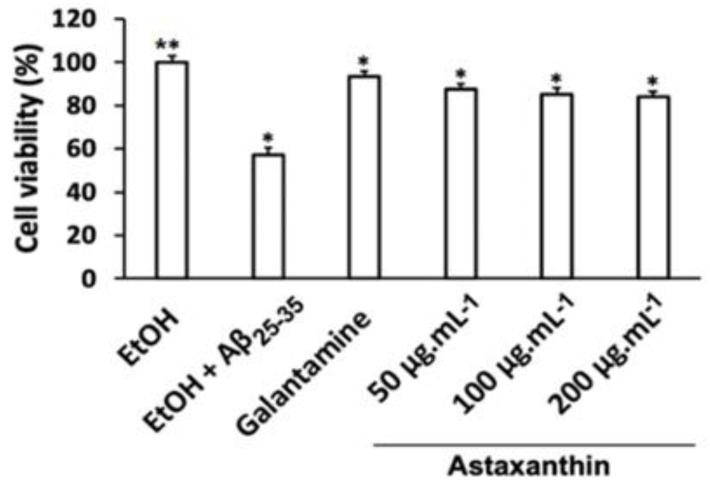
Neuroprotective effects of astaxanthin against Aβ_25–35_-induced neurotoxicity in C6 cell lines. Cells were pre-incubated with astaxanthin (at a concentration of 50, 100, and 200 µg mL^−1^) or galantamine (at 0.1 µg mL^−1^) for 24 h prior to 20 mM Aβ_25–35_ exposure for 1 h. Cell viability was assessed by an MTT assay. The data are expressed as the mean ± SEM (n = 3). Significant differences in the cell damage induced by Aβ_25–35_ are denoted by * *p* < 0.05; ** *p* < 0.01. EtOH: ethanol.

Treatment with Aβ_25–35_ reduced cell viability from 100% in the control group to 57.21% in the Aβ_25–35_-treated group (Figure 11). However, pre-treatment of C6 cells with astaxanthin at concentrations of 50, 100, and 200 µg mL^−1^, or with galantamine (0.1 µg mL^−1^), significantly increased cell viability to 87.72%, 85.16%, and 84.12%, respectively. These results indicate that astaxanthin exhibits neuroprotective effects in C6 cells subjected to Aβ_25–35_-induced cytotoxicity.

## 4. Discussion

*H. pluvialis* is known for its ability to accumulate astaxanthin, accounting for approximately 5% of its dry cell weight (DCW), making it a promising natural source of astaxanthin [21,30]. However, its slow growth, susceptibility to hydrodynamic stress, and morphological changes in response to environmental factors present challenges for large-scale cultivation. In this study, we demonstrated that the RM culture medium is the most effective and optimal medium for the growth of the *H. pluvialis* HB strain when compared to other tested media containing vitamins B_1_, B_12_, and H. The growth and cell density of *H. pluvialis* are influenced by several factors, including culture type, nutrient supply, and environmental conditions.

In the two-phase culture process, an increase in algal cell density, especially in the first phase, was observed. The availability of nitrate is crucial to maintaining *H. pluvialis* in its green vegetative state. This nutritional management is also key for maximizing astaxanthin accumulation. In the current study, a maximum cell density of 3.2 × 10^6^ cells mL^−1^ was achieved using an RM medium with a NaNO_3_ concentration of 1.2 g L^−1^ under a photoperiod of 16:8 h (light: dark) with 10 h of high-intensity light (85 µmol photons m^−2^ s^−1^) and 6 h of high white light combined with UV. Notably, increasing light intensity from 50 to 115 µmol photons m^−2^ s^−1^ and extending the lighting duration from 12 to 16 h, alongside UV irradiation, resulted in a significant rise in cell density (3.6-fold increase) from 0.9 × 10^6^ to 3.2 × 10^6^ cells mL^−1^.

Further improvements in cell density were observed with the use of the RM-4X medium. The perfusion culture method, involving the addition of a medium with a 10-fold higher nitrate concentration, supported the maintenance of green vegetative cells, reaching a maximum cell density of 4.96 × 10^6^ cells mL^−1^ after 22 days without morphological changes. This outcome surpasses the previously reported results [16,31,32], emphasizing the importance of nitrate concentration, light regimes, and nutrient supply in enhancing *H. pluvialis* growth.

The role of UV radiation in algal growth is noteworthy, particularly UV-A (320–400 nm), which can regulate photosynthesis and key metabolic enzymes, even in the presence of UV-B (280–315 nm). UV radiation, especially UV-A, has been shown to promote the growth of *H. pluvialis*, while high nitrate concentrations mitigate the negative effects of UV radiation by enhancing the recovery of vital metabolic enzymes [33,34].

Vitamin supplementation, particularly with B_1_, B_12_, and H, has also proven beneficial for optimal growth, which is consistent with previous studies [2]. The renewed media employed a perfusion culture technique, supporting higher cell densities while maintaining vegetative cell growth during the first phase of the culture. The density of vegetative cells in this study reached a maximum of 4.96 × 10^6^ cells mL^−1^, which is 25.7 times higher than that previously published by another author (19.3 × 10^4^ cell mL^−1^) [20].

Astaxanthin accumulation occurs when green vegetative cells of *H. pluvialis* transform into red cyst cells under stress conditions such as high light intensity, nitrogen limitation, and phosphate deprivation. Among these factors, nitrogen deprivation is crucial for inducing astaxanthin accumulation and is often regulated by the carbon-to-nitrogen (C/N) ratio [35]. Sodium bicarbonate (NaHCO_3_) with higher solubility in water compared to CO_2_ can be used as an inorganic carbon source [36]. When NaHCO_3_ dissolves in water, it makes HCO_3_^−^ ions available as the dissolved inorganic carbon source for microalgae. Sodium bicarbonate is being extensively tested as a “trigger” mechanism in lipid production. Since carotenoids are hydrophobic, pigments are dissolved and stored inside fatty acids, leading to an increase in pigment content as lipid production increases. *H. pluvialis* is the microalga known to produce the highest level of astaxanthin, and the reason may have to do with the efficient deposition of astaxanthin into lipid globules as esters so fatty acid metabolism may be correlated with pigment accumulation, which can be a main factor to enhance astaxanthin biosynthesis in algae cells.

Astaxanthin accumulation occurs when green vegetative cells of *H. pluvialis* transform into red cyst cells and is closely related to triglycerides (TAGs), which are stored in lipid bodies under stress conditions such as high light intensity, pH change, nitrogen limitation, and phosphate deprivation [37,38]. Among them, the carbon/nitrogen (C/N) ratio is an important factor in the accumulation of astaxanthin in *H. pluvialis.* Microalgae are capable of fixing carbon in the inorganic form of CO_2_ or bicarbonate (HCO_3_^−^) via photosynthesis, and increasing the C/N ratio by adding bicarbonate to the culture medium helps to accelerate the process and increase the efficiency of astaxanthin accumulation. Additionally, medium pH is also increased from 7 to 9 at an optimal concentration of NaHCO_3_. Adding a concentration of NaHCO_3_ combined with stress conditions, including high pH and nitrogen depletion, will stop cell growth, provide large amounts of bioavailable carbon, and, therefore, increase astaxanthin production. In our study, the most effective astaxanthin induction was achieved through nutrient deprivation and bicarbonate supplementation, with a high C/N ratio. Supplementing with 100 mM bicarbonate resulted in the rapid transformation of green cells into red cysts within just two days, yielding a maximum astaxanthin content of 48.8 mg g^−1^ DCW. This is the shortest induction time reported to date, suggesting potential for large-scale astaxanthin production in Vietnam [7,14].

While bicarbonate plays a crucial role as a carbon source, excessive concentrations may lead to increased alkalinity, which can reduce cell density but accelerate astaxanthin accumulation. The optimal bicarbonate concentration in our study was five times higher than that reported by Kang et al. [39], who found a maximum astaxanthin content of 29.7 mg g^−1^ biomass at 20 mM bicarbonate after 18 days of induction. The variation in the findings may be due to genetic differences between algal strains and culture environments.

In addition to astaxanthin’s potential for large-scale production, its neuroprotective effects were also evaluated in this study. Oxidative stress, a major contributor to neurodegenerative diseases, such as Alzheimer’s disease, can be mitigated by antioxidants, like astaxanthin [24]. Our results demonstrated significant antioxidant activity, with an IC_50_ value of 3.74 mg mL^−1^ for astaxanthin extracted from *H. pluvialis* HB, compared to 18.53 µg mL^−1^ for the positive control ascorbic acid. Astaxanthin also displayed acetylcholinesterase (AChE) inhibitory activity, with an IC_50_ value of 297.99 µg mL^−1^, which is comparable to galantamine (4.11 µg mL^−1^), a standard treatment for Alzheimer’s disease.

Moreover, astaxanthin exhibited neuroprotective properties against oxidative stress and Aβ_25–35_-induced toxicity in C6 cells. These results align with previous studies [2,13], and this study is the first to report on the neuroprotective activity of astaxanthin from the *H. pluvialis* HB strain.

## 5. Conclusions

In conclusion, *H. pluvialis* HB from Vietnam is a promising source for commercial astaxanthin production. Our study achieved high-density cultivation (4.96 × 10^6^ cells mL^−^^1^) using a cost-effective perfusion culture method with optimized nitrate and light conditions. Astaxanthin accumulation reached 48.8 mg g^−1^ DCW in just two days under nutrient deprivation and bicarbonate supplementation. The extracted astaxanthin showed no cytotoxicity up to 200 μg mL^−^^1^ and demonstrated strong antioxidant and neuroprotective activities, highlighting its potential for large-scale production and use in neuroprotective health products. Nutrient deprivation followed by supplementation with 100 mM bicarbonate and constant aeration resulted in a maximal astaxanthin concentration of 48.8 mg/g of dry cell weight within two days, indicating that this approach is feasible and effective for large-scale astaxanthin synthesis. The obtained results will provide new applications of astaxanthin in the prevention and treatment of neurodegenerative diseases and investigate the most potential novel astaxanthin delivery system in clinical trials. An additional consideration that must be taken into account for future study is the mechanism of action or pharmacokinetics of astaxanthin as a neuroprotective agent, and its benefits must be elucidated in the production of health supplements or drugs in the treatment of neurodegenerative diseases, such as Alzheimer’s disease. Additional studies are needed to elucidate the precise pathophysiological pathways involved in neurodegeneration and to clarify the potential neuroprotective effects of astaxanthin extracted from *H. pluvialis* HB biomass on humans.

## Figures and Tables

**Table 1:** Antioxidant activity of astaxanthin isolated from *H. pluvialis* HB compared with the control, ascorbic acid.

DPPH Scavenging Activity (%)		DPPH Scavenging Activity (%)	
Concentration (mg mL^−1^)	Astaxanthin	Concentration (µg mL^−1^)	Ascorbic Acid
0.1	7.39 ± 0.13	4	30.30 ± 0.43
0.4	12.87 ± 0.09	20	65.05 ± 1.23
2	29.87 ± 0.54	100	91.45 ± 1.34
IC_50_ (mg mL^−1^)	3.74 ± 0.25	IC_50_ (µg mL^−1^)	18.53 ± 0.97

Ascorbic acid: positive control; astaxanthin isolated from *H. pluvialis* HB. Antioxidant activity of astaxanthin and ascorbic acid determined by the DPPH method.

**Table 2:** Acetylcholinesterase inhibitory activities of astaxanthin.

AChE Inhibitory Activity (%)		AChE Inhibitory Activity (%)	
Concentrations (µg mL^−1^)	Astaxanthin	Concentrations (µg mL^−1^)	Galantamine
4	3.21 ± 0.09	0.08	8.98 ± 0.45
20	20.87 ± 0.78	0.4	24.89 ± 1.21
100	45.21 ± 1.12	2	52.78 ± 1.34
500	68.76 ± 3.76	10	86.56 ± 1.98
IC_50_ (µg mL^−1^)	297.99 ± 5.23	IC_50_ (µg mL^−1^)	4.11 ± 0.25

## Data Availability

All data were presented in the manuscript.

## Supplementary Materials

The following supporting information can be downloaded at https://www.mdpi.com/article/10.3390/bioengineering11121176/s1, Supplemental Figure S1. Cell density of *Haematococcus* sp. in the RM medium. Supplemental Figure S2. Astaxanthin content of *H. pluvialis* HB at different illumination conditions. Supplemental Figure S3. Culture flasks of *H. pluvialis* HB in the second phase—induction of astaxanthin accumulation by the difference in HCO_3_^−^ concentrations. Supplemental Figure S4. Cell morphological changes of *H. pluvialis* HB in the second phase—induction of astaxanthin accumulation by adding 100 mM HCO_3_^−^. Supplemental Figure S5. Cultivation of *H. pluvialis* HB in a 20 (A), 50 (B), and 100 L (C) bioreactor in the second phase when inducing astaxanthin accumulation by 100 mM HCO_3_^−^.
