# Supplementary material for: Optimization of Culture Conditions for High Cell Productivity and Astaxanthin Accumulation in Vietnam’s Green Microalgae Haematococcus pluvialis HB and a Neuroprotective Activity of Its Astaxanthin
Source: Bioengineering (Basel). 2024 Nov 21;11(12):1176. doi: 10.3390/bioengineering11121176 (PMC11672925; doi:10.3390/bioengineering11121176)
Supplement: Supplementary file 1 [file bioengineering-11-01176-s001.zip › bioengineering-3280182-supplementary.pdf]

Supplemental S

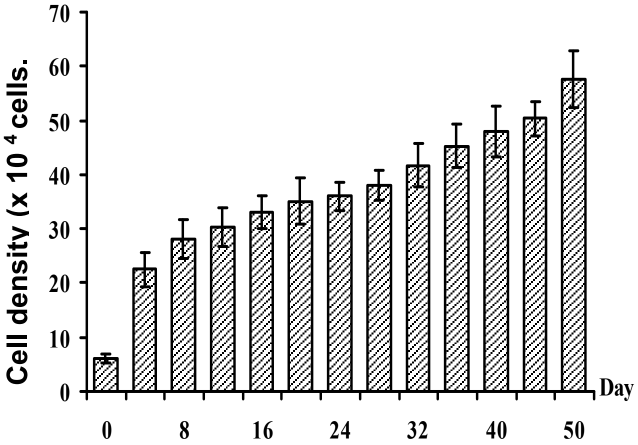

Supplemental Figure S1. Cell density of *Haematococcus* sp. in RM medium

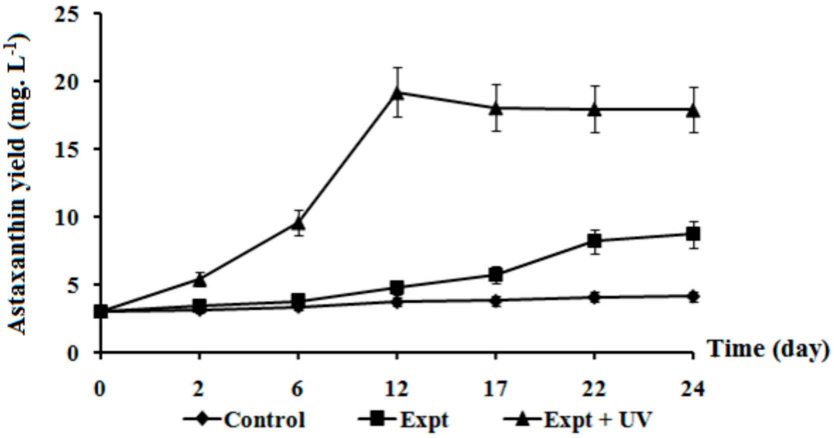

Supplemental Figure S2. Astaxanthin content of *H. pluvialis* HB at different illumination conditions

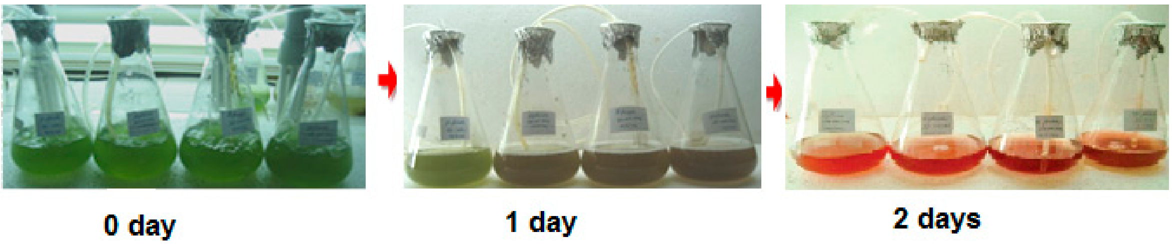

Supplemental Figure S3. Culture flasks of *H. pluvialis* HB in second phase - Induction of astaxanthin accumulation by difference HCO_3^- concentration

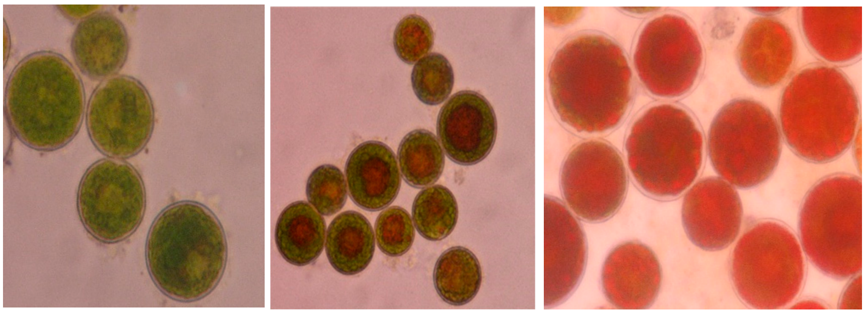

0 day **1 day** **2 days**

Supplemental Figure S4. Cell morphological changes of *H. pluvialis* HB in the second phase - Induction of astaxanthin accumulation by adding 100 mM HCO_3^-

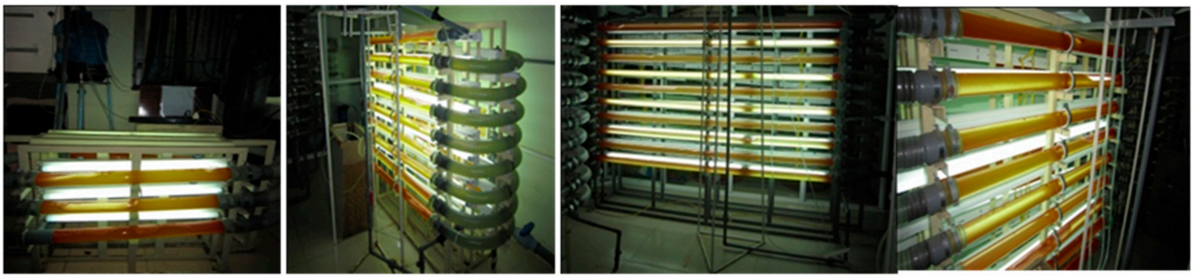

A **B** **C**

Supplemental Figure S5. Cultivation of *H. pluvialis* HB in 20 (A), 50 (B) and 100 L (C) bioreactor in the second phase when inducing astaxanthin accumulation by 100 mM HCO_3^-
